# Renal globotriaosylceramide deposits for Fabry disease linked to uncertain pathogenicity gene variant c.352C>T/p.Arg118Cys: A family study

**DOI:** 10.1002/mgg3.981

**Published:** 2019-09-30

**Authors:** Magdalena Cerón‐Rodríguez, Guillermo Ramón‐García, Edgar Barajas‐Colón, Isidro Franco‐Álvarez, Juan L. Salgado‐Loza

**Affiliations:** ^1^ Department of Lysosomal Diseases Hospital Infantil de México Federico Gómez Mexico City México; ^2^ Department of Pathology Hospital Infantil de México Federico Gómez Mexico City México; ^3^ Department of Nephrology Hospital Infantil de México Federico Gómez Mexico City México; ^4^ SICODIC Ciudad de México México

**Keywords:** beta agalsidase, c.352C>T/p.Arg118Cys variant, children, fabry disease, renal Gb3 deposits

## Abstract

**Background:**

Fabry disease (FD) has an extensive phenotypic expression associated with *GLA* gene variants. The *GLA* gene variant c.352C>T/p.Arg118Cys was considered with uncertain pathogenicity because of the finding of high residual alpha‐galactosidase A (α‐Gal A) enzyme activity, the absence of Mendelian segregation with an FD phenotype with many individuals remaining asymptomatic at old ages and the lack of globotriaosylceramide (Gb3) deposits in tissues. Gb3 deposits are found in kidneys before the progression to overt microalbuminuria and decreased glomerular filtration.

**Methods:**

We describe a family with c.352C>T/p.Arg118Cys variant and pathognomonic signs of FD renal damage in masculine children.

**Results:**

The proband died of end‐stage renal failure and we analyzed *GLA* gene in his offspring and found the variant in all daughters and five of seven grandchildren. In patients who we measure plasma and urinary Gb3, α‐Gal A enzyme activity, and plasma globotriaosylsphingosine (Lyso‐Gb3), these were normal or almost normal. A kidney biopsy was performed in two boys and one girl with normal renal function and characteristic signs of FD as enlarged and vacuolated epithelial cells, myelin figures, myelin‐like figures, lamellated structures in podocytes and endothelial cells, were found in boys. These boys received agalsidase beta 1 mg/kg IV infusion every other week to prevent further renal damage.

**Conclusion:**

This is the first report that shows a link between FD renal Gb3 deposits and c.352C>T/p.Arg118Cys variant, supporting pathogenicity of a variant considered until now with uncertain pathogenicity.

## 1 INTRODUCTION

Fabry disease (FD, OMIM # 301500), is an X‐linked disease caused by variants in the *GLA* gene (GenBank reference sequence NG_007119.1), located at locus Xq22.1, producing deficient hydrolysis activity of α‐galactosidase A enzyme (α‐Gal A; E.C. 3.2.1.22), which leads to progressive lysosomal accumulation of globotriaosylceramide (Gb3, ChemIDplus 0071965576) and globotriaosylsphingosine (lyso‐Gb3, ChemIDplus 0126550865) in cells and body tissues (Desnick, Ioannou, & Eng, 2001). Fabry disease has an extensive phenotypic expression associated with different *GLA* gene variants; more than 800 variants are pathogenic and expressed in men with α‐Gal A activity <1% as the classic form of FD. When α‐Gal A activity is >1%, men can express late‐onset cardiac or renal FD phenotypes or a cerebrovascular disease presenting as stroke or transient ischemic attack (Mehta & Hughes, 2002, updated in 2017). Lukas et al proposed four classes of mutations based upon the enzyme activity data: class I: 0%, class II: >0%–20%, class III: ≥20%–60%, class IV: ≥60% and found that 42 class I variants with 0% enzyme activity were linked to classic FD phenotype while class III and IV variants with activity greater than 20% tended to late‐onset FD (Lukas et al., 2013). Heterozygous women typically have milder symptoms than men but may have symptoms as severe as those observed in men (Deegan, Bähner, Barba, Hughes, & Beck, 2006 2006). Other variants linked to FD are classified as benign (c.937G>T/p.Asp313Tyr, rs28935490) or with uncertain pathogenicity (c.352C>T/p.Arg118Cys, rs148158093 and c.427G>A/p.Ala143Thr, rs104894845) because they were associated with residual activity of α‐Gal A of about 20% without clear manifestations of FD (Mehta & Hughes, 2002, updated in 2017).

Although renal disease in classic or renal late‐onset phenotype FD is more common in adults, it has also been reported in children and renal failure observed as early as 16 years of age (Sheth, Roth, & Adams, 1983). Light and electron microscopy studies of renal biopsies in nine FD symptomatic men and women aged 7 to 18 years showed podocyte inclusions, segmental foot process effacement, and distal tubular inclusions in all patients in spite of normal glomerular filtration and albuminuria in five, confirming that deposition of Gb3 starts in early childhood before overt renal disease (Tøndel, Bostad, Hirth, & Svarstad, 2008).

We describe a family with c.352C>T/p.Arg118Cys variant and pathognomonic signs of FD renal damage in masculine children. This is the first report that shows objective evidence of FD renal Gb3 deposits linked to c.352C>T/p.Arg118Cys variant.

### 1.1 Family case

Ethical Compliance: this study was approved by ethics committee. Female patients and the parents of the children were informed about the study procedures and treatment. Informed consent was obtained.

**Figure 1:**
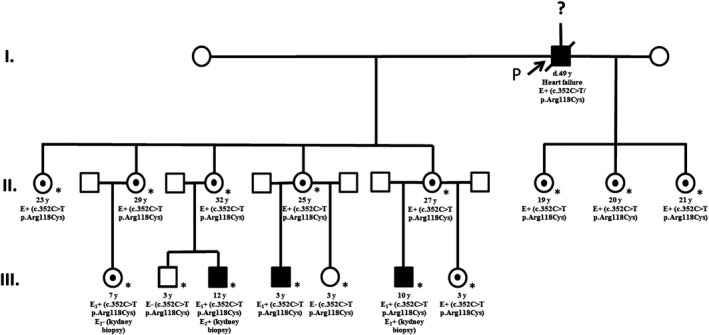
Genogram of c.352C>T/p.Arg118Cys variant family. Circles indicate females, squares indicate males. Black squares are the hemizygous affected male members and black pointed circles indicate female heterozygous patients. ? = family history not available. P = proband. E + or E1+ = positive for variant. E‐ = negative for variant. E2+ = kidney biopsy positive for Gb3 deposits. E2− = kidney biopsy negative for Gb3 deposits. d. = death age. * = documented evaluation

**Figure 2:**
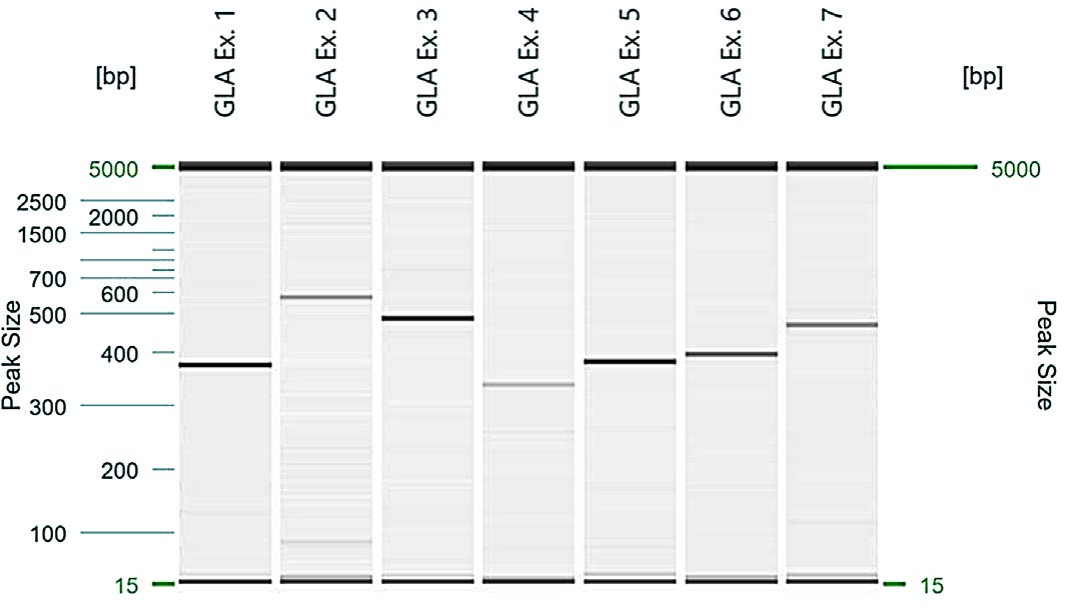
Patient 2. PCR amplification of all seven exons of the *GLA* gene showing the absence of deletion.. (Courtesy of Laura Pollard, PhD, FACMG, Greenwood Genetic Center, Greenwood SC, USA)

The proband case was a 49‐year‐old man with idiopathic end‐stage renal failure with hemodialytic treatment from four years before, enrolled in an open screening study of 200 patients with chronic kidney disease treated in a hemodialysis unit independently of the initial diagnosis to discard uncommon etiologic causes of renal damage. The expected prevalence of FD was 1 in 200 (0.500% CI 95%, 0.000%, 1.478%). The screening analyzed *GLA* gene by sequencing of DNA strands of the coding region and c.352C>T/p.Arg118Cys variant in exon 2 was found. No more data of this analysis were available. It was not possible to measure α‐Gal A activity or Gb3 levels because shortly after the proband died as a result of heart failure secondary to end‐stage renal failure. The diagnosis of probable FD in proband was made because the finding of variant and the history of renal failure. Then to establish the variant presence and Gb3 levels, we performed *GLA* gene sequencing, plasmatic Gb3 (pGb3) and urinary Gb3 (uGb3) in the offspring (Figure 1). The presence of the variant in proband's mother or other relatives is unknown because upwards variant screening was not possible to do. The proband was an only child and ignore if other relatives were affected. In all daughters of two marriages (eight), a heterozygous c.352C>T/p.Arg118Cys variant in exon 2 was found and the levels of pGb3 and uGb3 were normal in all of them (Table 1). All except one were asymptomatic and without renal, cardiac, or neurological damage. The symptomatic daughter only had varicose pain. Four of these women had seven children: four boys and three girls. We determined *GLA* gene targeted sequencing in all children. The c.352C>T/p.Arg118Cys in exon 2 variant was found in five children: three boys (patients 1, 2, and 3) and two girls (patients 5 and 6), no other alteration was found in the amplification by PCR and subsequent analysis with standard fluorescent sequencing protocol in both forward and reverse direction of all seven coding exons of the *GLA* gene and immediate flanking intron sequences (Figure 2) (Greenwood Genetic Center, Greenwood SC, USA); α‐Gal A activity, pGb3, uGb3, and Lyso‐Gb3 were measured in patients 1, 2, and 6 and α‐Gal A activity in patients 3 and 5. Patient 1 had growth failure associated with bad eating habits; patient 2 referred acroparesthesias and diarrhea 2–3 times a month and patients 3, 5, and 6 were asymptomatic. The variant was not found in one boy and one girl (patients 4 and 7) (Table 2).

**Table 1:** Plasma and Urine Gb3 of proband's daughters with heterozygous c.352C>T/p.Arg118Cys variant

Pat	Age	Plasma Gb3 (μg/ml) nl: <7.0	Urine Gb3 (μg/mmol creatinine) nl: <81
1	28	2.7	BQL
2	23	3.7	BQL
3	20	2.3	BQL
4	27	2.3	BQL
5	25	3.1	BQL
6	29	2.8	BQL
7	21	3.6	BQL
8	19	2.5	BQL

Abbreviations: Pat, patient; BQL, Below quantity level; Gb3, globotriaosylceramide; nl, normal.

**Table 2:** Characteristics of children with c.352C>T/p.Arg118Cys variant

Pat	Age	Sex	*GLA* gene variation	α‐Gal A activity (μmol/L/hr) nl: >1.64	Plasma Gb3 (μg/ml) nl: <7.0	Urine Gb3 (μg/mmol creatinine) nl: <81	Plasma Lyso‐Gb3 (ng/ml) nl: <5.0	Biopsy
1	12	boy	Hemizygous exon 2 c.352C>T/p.Arg118Cys rs148158093	3.01	BQL	BQL	BQL	Yes
2	10	boy	Hemizygous exon 2 c.352C>T/p.Arg118Cys rs148158093	1.61	3.1	BQL	BQL	Yes
3	3	boy	Hemizygous exon 2 c.352C>T/p.Arg118Cys rs148158093	2.01	Na	Na	na	No
4	11	boy	None	Na	Na	Na	na	No
5	3	girl	Heterozygous exon 2 c.352C>T/p.Arg118Cys rs148158093	Na	Na	Na	Na	No
6	7	girl	Heterozygous exon 2 c.352C>T/p.Arg118Cys rs148158093	3.5	BQL	BQL	BQL	Yes
7	3	girl	None	Na	Na	Na	Na	No

Abbreviations: BQL, Below quantity level; na, not available; α‐Gal A, α‐galactosidase A enzyme; Gb3, globotriaosylceramide; Lyso‐Gb3, globotriaosylsphingosine; Pat, patient; nl, normal.

Cardiac, neurological, ophthalmological, audiological, and renal evaluations were made in all children with the variant. Neurological, ophthalmological, and audiological evaluations were normal in all of them. Cardiac evaluation only shows tricuspid insufficiency and right bundle branch block in patient 1, other patients were normal. The renal evaluation shows normal renal function, normal plasmatic creatinine level, and absence of microalbuminuria in all of them.

We decided to carry out an ultrasound‐guided percutaneous kidney biopsy with BARD® MAGNUM® reusable biopsy gun system (BD Bard, Covington, USA) and BARD® MAGNUM® 16G needle (BD Bard) directed to the lower pole of left kidney with posterior hemostasis to obtain specimens for light and electron microscopic analysis, in older affected children (patients 1, 2, and 6), even with the absence of microalbuminuria to discard renal damage because of grandfather history, the antecedent of another asymptomatic and almost normal ∝‐GAL A activity patient with a reported pathogenic variant and renal damage (data not published) and previous report of glomerular and vascular changes before overt microalbuminuria in children with FD (Tøndel et al., 2008). We inform the parents about the lack of criteria to perform the procedure and TRE benefits if a positive biopsy was found to avoid further kidney damage and they give us their consent.

After the biopsy, the specimens were placed on a slide with 0.9% saline solution and two minutes after they were evaluated with light microscopy to search for glomeruli. A biopsy with eight or more glomerulus was considered useful for analysis. The tissue for light microscopic analysis was fixed with 10% formaldehyde buffer, embedded in standard histological grade paraffin, and 4 μm thick cuts with Leica microtome (Leica, Wetzlar, Germany) were obtained for Hematoxylin‐Eosin (H‐E), periodic acid‐Schiff (PAS), Masson trichromic (Masson), and silver methenamine (Jones) stains. For electronic microscopy analysis, a sample of fresh tissue was fixed with 2.5% glutaraldehyde in cacodylate buffer followed by phosphate buffer solution (PBS) and postfix with 2% osmium tetraoxide for membranes and 4% uranyl acetate for nucleic acid. Once the tissue has been fixed, it was gradually dehydrated with 30%, 50%, 60%, 70%, 80%, and 90%, absolute ethanol and propylene oxide ACS reagent 20,401, and then it was embedded in epoxy resin and polymerized at 60°C for 24 hr. We obtained 200–350 nm semi‐thin cuts with Leica RM2155 sf rotary microtome (Leica, Wetzlar, Germany) with glass knife for toluidine blue stain. Stained cuts were evaluated with light microscopy to select fields for 60–90 nm ultrathin cuts with Leica RM2155 sf rotary microtome (Leica, Wetzlar, Germany) with Diatome diamond knife (Diatome, Hatfield, USA) and reviewed with JEOL JEM‐1010 transmission electron microscope (Jeol Ltd, Tokyo, Japan).

**Figure 3:**
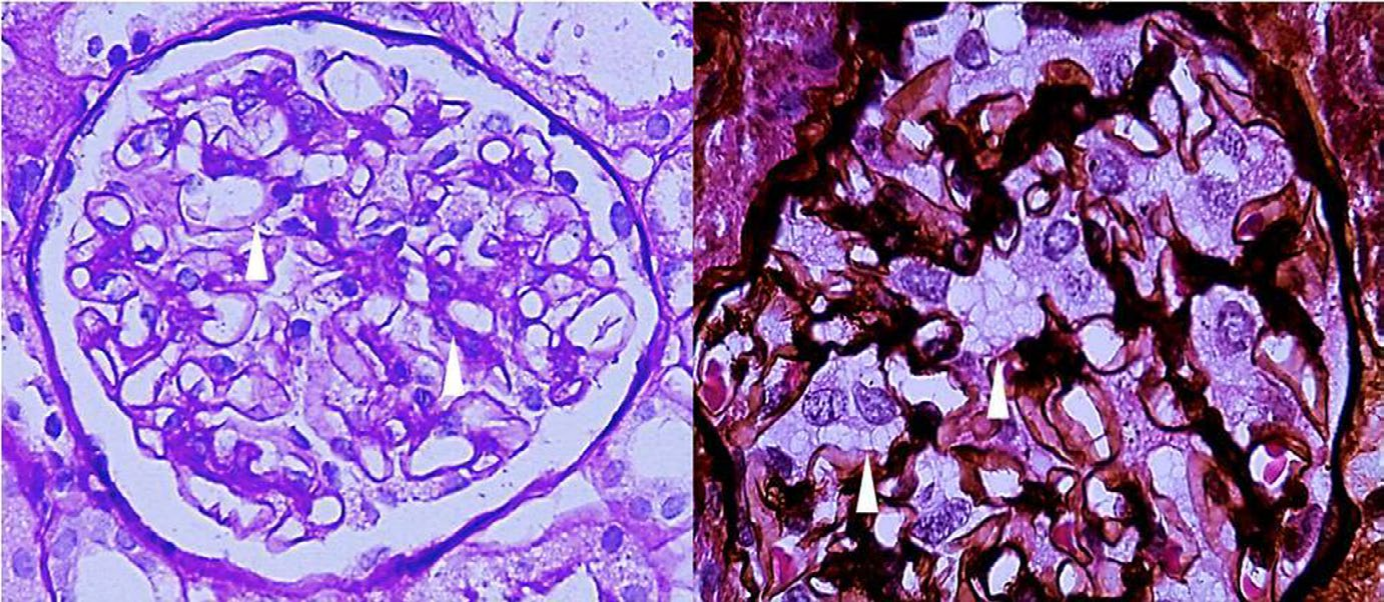
Patient 1. Left: Glomerulus with visceral epithelial cells showing cytoplasmic expansion that becomes vacuolated (arrow) (PAS 40×). Right: Glomerulus with visceral epithelial cells showing clear cytoplasmic expansion and vacuoles (arrow) (silver methenamine of Jones 40×)

**Figure 4:**
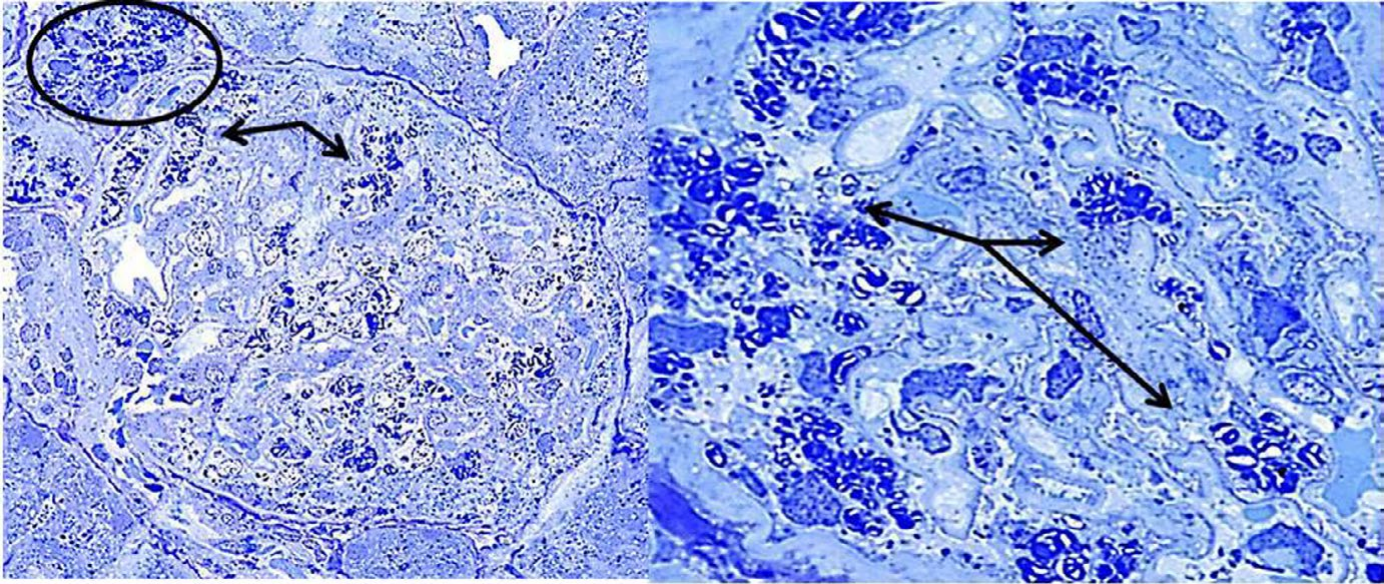
Patient 1. Toluidine blue‐stained semi‐thin section showing abundant inclusions in >50% of podocytes with cytoplasmic expansion (arrows), some inclusions in endothelial and mesangial cells and scarce inclusions in distal tubules (oval). Left: 40×, Right: close up of 40×

**Figure 5:**
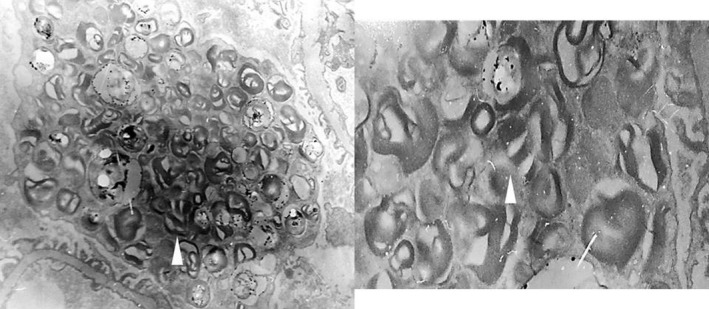
Patient 1. Left: Electronic microscopy showing podocytes with myelin figures and zebra bodies in cytoplasm (arrow) (4,500×). Right: Zebra bodies (arrow) (close up of 4,500×). All podocytes had lamellated inclusions, some with more inclusions than others

**Figure 6:**
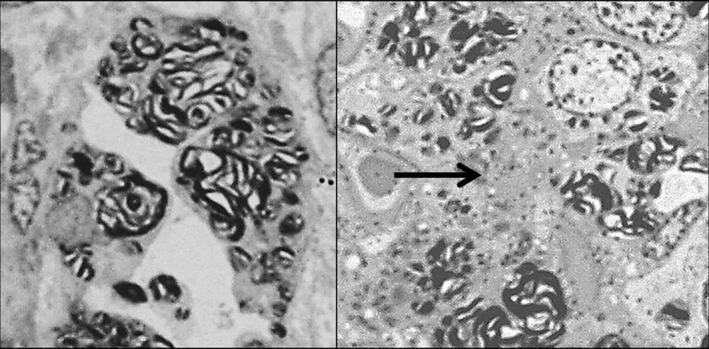
Patient 1. Left: lamellated and zebra bodies in capillary endothelial cell (4,000×). Right: abundant lamellated and zebra bodies in epithelial cells and some in mesangial cells (arrow) (2,500×)

In patient 1, light microscopy of renal biopsy with H‐E, PAS, Masson and Jones stains identified 15 normal size glomeruli with a mild increase in mesangial cellularity, large visceral epithelial cells with cytoplasmic expansion that becomes vacuolated, tubules with swelling and scarce hyaline cylinders, normal endothelial cells and normal capillary loops (Figure 3). Toluidine blue‐stained semi‐thin section showed abundant inclusions in >50% of podocytes with cytoplasmic expansion, some inclusions in endothelial and mesangial cells and scarce inclusions in distal tubules (Figure 4). Electron microscopy of renal biopsy identified vacuoles and few concentrically laminated electron‐dense inclusions with myelinic appearance in endothelial cell and epithelial cells had few concentrically laminated inclusions, myelin figures, and zebra bodies (Figures 5 and 6). The basement membrane was normal with uniform thickness and without evidence of electron‐dense deposits or other alterations and mild increase in mesangial matrix with some mesangial cell with few concentrically laminated electron‐dense inclusions was found. The extension of glycosphingolipid storage by cell type was: >50% podocyte, 5% mesangial cells, 10% endothelial cells, 5% distal tubules affected and a few proximal tubules affected. Scoring system for renal pathology in Fabry disease (Fogo et al., 2010): light microscopy: 15 glomeruli. Mean podocyte score: 7.46, without other glomerular (sclerosis, nonsclerotic, adhesion, ischemic, fibrosis or reduplication), arterial (sclerosis or hyalinosis), or tubulointerstitial (fibrosis or inflammation) lesions. Thick section: 3 glomeruli without global or segmental sclerosis, podocyte inclusions 1+ ‐ 2+ and presence of inclusions in parietal epithelial, proximal, and distal tubular cells.

**Figure 7:**
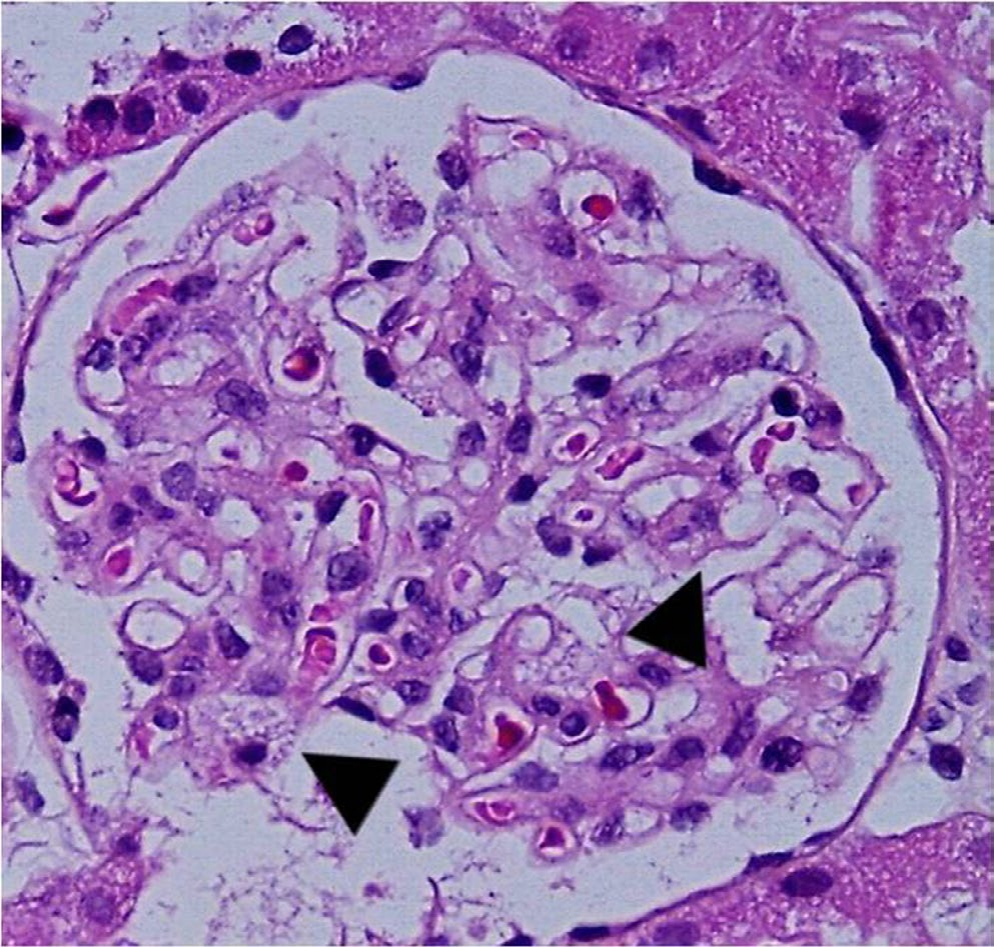
Patient 2. Glomerulus with minimal mesangial proliferation and vacuolated visceral epithelial cells (arrow) (Hematoxylin‐Eosin 40×)

**Figure 8:**
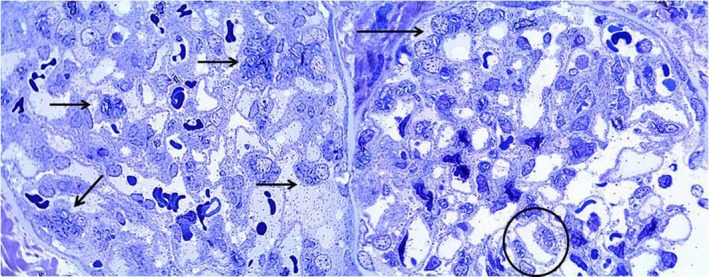
Patient 2. Left: Toluidine blue‐stained semi‐thin section showing inclusions in 30% of podocytes (arrows) (40×). Right: Toluidine blue‐stained semi‐thin section showing inclusions in 30% of podocytes (arrows) and scarce inclusions in endothelial cells (circle) (40×)

**Figure 9:**
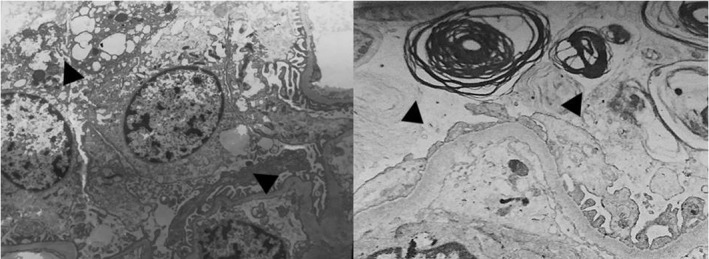
Patient 2. Left: Glomerulus with vacuolated visceral epithelial cell (arrow) (2000×). Right: Laminated figures in visceral epithelial cell (arrow) (4,000×)

In patient 2, light microscopy of renal biopsy with H‐E, PAS, Masson and Jones stains showed 18 glomeruli with mild mesangial proliferation, prominent podocyte with vacuoles, tubules with droplets of resorption and normal vascular and tubulointerstitial tissues (Figure 7), Toluidine blue‐stained semi‐thin section showed inclusions in 30% of podocytes and scarce inclusions in endothelial cells (Figure 8). Electron microscopy of renal biopsy identified epithelial cells with large, prominent and abundant vacuoles and wide cytoplasm with scarce myelinic bodies (Figure 9). Podocytes have normal pedicels with foci of segmental fusion. The basement membrane was trilaminar without thickness alterations and homogeneous dense sheet. Endothelial cells were prominent and have fenestration preservation with some intracytoplasmic tubuloreticular inclusions. The mesangium had an increase in mesangial matrix and the number of mesangial cells. The extension of glycosphingolipid storage by cell type was: 30% podocyte, 0% mesangial cells, 5% endothelial cells, 2% distal tubules affected, and no proximal tubules affected. Scoring system for renal pathology in Fabry disease (Fogo et al., 2010): light microscopy: 18 glomeruli. Mean podocyte score: 0.5, without other glomerular (sclerosis, nonsclerotic, adhesion, ischemic, fibrosis, or reduplication), arterial (sclerosis or hyalinosis), or tubulointerstitial (fibrosis or inflammation) lesions. Thick section: 2 glomeruli without global or segmental sclerosis, podocyte inclusions 1+ and without inclusions in parietal epithelial, proximal and distal tubular, peritubular capillary, vascular intimal, or medial cells.

In both patients 1 and 2, podocyte vacuolation was observed in all glomeruli but it was greater in some than others and correlates with lamellated inclusion in electronic microscopy examination.

In patient 6, renal biopsy with H‐E, PAS with/without diastase, Masson and Jones stains showed 15 normal glomeruli and we diagnosed it as normal without changes in renal tissue. No electron microscopy analysis was made.

In patients 1 and 2, we started enzymatic replacement therapy (ERT) with agalsidase beta at a dose of 1 mg/kg IV infusion every other week (Fabrazyme; Genzyme, Cambridge, MA, USA) and now they have 38 and 28 months of treatment, respectively. They remain asymptomatic with normal renal function and we will do a new biopsy after 60 months of treatment. Patient 5 is under observation without treatment. We will make a biopsy in patients 3 and 6 as soon as they are old enough to perform it and if they have kidney FD changes we will start ERT.

We defined the carrier status in all women because of the absence of symptoms, normal renal function and normal activity of pGb3 y uGb3.

## 2 DISCUSSION

FD has a wide range of manifestation related to several gene variants, a lot of them linked to a specific kind of pathologic patterns. Spada (Spada et al., 2006), described c.352C>T/p.Arg118Cys variant in one neonate with α‐GAL A activity in plasma of 0.7 U/ml (normal level 14.3 ± 3.2 U/ml). After, different publications have described this variant in young patients with stroke (Baptista et al., 2010; Rolfs et al., 2013), cardiac myopathy (Elliot et al., 2011), and renal failure (Gaspar et al., 2010). These studies were intrinsically biased because they studied high‐risk patients with multifactorial caused conditions instead of an open population (Ferreira et al., 2015), nevertheless, all of them confirm a relationship between c.352C>T/p.Arg118Cys variant and organ damage but none of them found GB3 deposits (Baptista et al., 2010; Elliot et al., 2011; Gaspar et al., 2010; Rolfs et al., 2013). The prevalence of c.352C>T/p.Arg118Cys variant in stroke reported by PORTYSTROKE study (Baptista et al., 2010), was 1.4% (6/493 patients, total prevalence 2.4%), while SIFAP study (Rolfs et al., 2013), reported a prevalence of 1% and mention the presence of c.352C>T/p.Arg118Cys variant in one patient at least. In cardiac myopathy c.352C>T/p.Arg118Cys variant was found in one female patient (total allelic variants prevalence 0.5% 7/1386 patients) (Elliot et al., 2011), and Gaspar found c.352C>T/p.Arg118Cys variant in 4 of 911 patients with renal failure in hemodialytic treatment (overall prevalence 0.55% 8/911 patients) (Gaspar et al., 2010). The Cys118 allele has been associated with high residual α‐Gal activity in vitro, as happened with our patients 1, 2, 3, and 6, and classified as a pathogenic variant, mainly on the theoretical basis of the chemistry of the cysteine residue, but has never been demonstrated by pathology criteria (Ferreira et al., 2015). It should be noted, however, that the correlations between the in vitro α‐Gal residual activity, substrate accumulation, and the FD clinical phenotype are complex and still incompletely understood, and that other factors, besides the residual level of enzyme activity, play a crucial role in the pathogenesis of the disease (Weidemann et al., 2013). It might also be possible that the in vitro α‐Gal assays do not reflect the biological enzyme activity in vivo, thereby confounding the interpretation of genotype‐phenotype correlations (Ferreira et al., 2015). Lukas et al found that overexpressed mutant α‐Gal A was abundantly present in the cells, notably above the endogenous level at the expected size of 46 kDa (Lukas et al., 2013), then the protein was evidently processed (Desnick et al., 2001), but it is not catalytically active and p.R118 allele maintain enzyme activity between 20% and 80% of the wild type enzyme and also have lower and similar to normal lisoGb3 values (Lukas et al., 2013). The assumption that *GLA* p.(Arg118Cys) is a pathogenic variation causing a later‐onset FD phenotype was based on theoretical considerations about the similarities of the structural changes it induces in the α‐Gal monomer and of its in vitro overexpression levels, with those of well‐known missense *GLA* variations associated with later‐onset clinical phenotypes, as well as on the reasoning that its sulfhydryl‐binding potential might interfere with the normal disulfide bonds of the α‐Gal monomers (Spada et al., 2006). Ferreira et al. (2015), concluded that based upon detailed and unbiased clinical, biochemical, histopathologic and family data, the mild/moderate deficiency of α‐Gal activity associated with c.352C>T/p.Arg118Cys is not of enough magnitude to cause major complications of FD and Arends (Arends et al., 2017), consider c.352C>T/p.Arg118Cys variant as a neutral (non‐pathogenic) variant because of the absence of characteristic storage in relevant organs and non‐producer of FD; then enough clinical, biochemical, and histopathological details to support the diagnosis of FD should be demonstrated (Ferreira et al., 2015).

Perhaps, previous reports seem conclusive to demonstrate non‐pathogenicity linked with c.352C>T/p.Arg118Cys variant, we decided to perform biopsies in our patients to discard Gb3 deposits in kidneys associated with c.352C>T/p.Arg118Cys variant based in the familiar history of a severe and apparent idiopathic renal failure developed by proband in his 49 years of life, the assumption that c.352C>T/p.Arg118Cys is a pathogenic variant causing a later‐onset FD phenotype (Spada et al., 2006), the existence of other reported patients with renal failure with this variant (Gaspar et al., 2010), and the fact of glomerular and vascular changes before the progression to overt microalbuminuria and decreased glomerular filtration rate in children with FD reported by Tøndel et al. (2008) and Wijburg et al. (2015), The specimens of patients 1 and 2 showed enlarged and vacuolated glomerular visceral epithelial cells, abnormal tubules and blood vessels in light microscopy, and zebra bodies, myelin figures, myelin‐like figures or lamellated structures in podocytes and endothelial cells in electron microscopy that are classical renal changes of FD (Sunder‐Plassmann, 2006), and support, for the first time in literature, c.352C>T/p.Arg118Cys variant pathogenicity by pathological criteria. Recently, Barbeito‐Caamaño et al. (2018), reported a family with p.Arg118Cys variant but deletion of exons 3 and 4 (p.Val124_Lys213del) was also present. She found cardiac and renal involvement in nine members with zebra bodies in kidney biopsy of one of them. In our family, all seven exons and immediate flanking intron regions were amplified by polymerase chain reaction, studied by standard fluorescent sequencing protocol in both forward and reverse direction, and detection of single nucleotide changes and small deletion or insertion examined (Greenwood Genetic Center, Greenwood SC, USA). Our patients only had p.Arg118Cys without deletions or other variations in *GLA* gene, then the Gb3 deposits, different from the family studied for Barbeito‐Caamaño et al. (2018), can only be associated with p.Arg118Cys variant. We examined our patients with a Sanger type sequencing while Barbeito‐Camaño analyzed her family with next‐generation sequencing, so unidentified pathogenic variants as deep intronic variants, might not be ruled out in our family. However, Sanger sequencing has high sensitivity to detect single nucleotide changes and small deletions or insertions (Gomes & Korf, 2018).

With the finding of Gb3 deposits in renal tissues and c.352C>T/p.Arg118Cys variant presence in our two boy patients, we started ERT with agalsidase beta 1 mg/kg every other week (Fabrazyme; Genzyme, Cambridge, MA, USA) even if they did not have microalbuminuria or functional evidence of kidney damage to prevent renal damage progression, based on the articles by Thurberg (Thurberg et al., 2002) that used ERT with agalsidase beta 1mg/kg every other week and resulted in a significant clearance of accumulated substrate in all cell types involved in renal FD that may halt pathology progression and prevent renal failure; Tøndel (Tøndel et al., 2013), that described a treatment‐associated prevention of progressive kidney disease in young patients with early FD nephropathy that can be achieved as a result of the potential reversibility of early functional and structural renal cell changes because early initiation of treatment and the use of sufficiently high enzyme dose can reach maximal clearance of Gb3 in kidney cells; and Wijburg (Wijburg et al., 2015), that found histologic evidence of Gb3 accumulation and cellular and vascular injury in renal tissues, associated to pathogenic variants at very early stages of the disease before the onset of microalbuminuria and the development of clinically significant renal events in children, supporting early initiation of enzyme replacement therapy to potentially improve long‐term outcome.

It is important to remember that symptoms may appear late in the curse of the disease when the organ damage is severe or not reversible and the value of the ERT is less. So consider an active screening to find other FD affected family members, to have the opportunity to treat them early if they have a pathological variant, to avoid early and late kidney or other organ damage developed for Gb3 deposits becomes transcendent especially in children.

This is the first report that shows a link between FD renal Gb3 deposits and c.352C>T/p.Arg118Cys variant, supporting pathogenicity of a variant considered until now as non‐pathogenic.

## CONFLICT OF INTEREST

Magdalena Cerón‐Rodríguez received reimbursement for attending a symposium and a fee for speaking from Sanofi Genzyme. Juan L Salgado‐Loza received payment for writing assistance from Sanofi Genzyme. Edgar Barajas‐Colón, Guillermo Ramón‐García and Isidro Franco‐Álvarez do not have competing interest. The authors confirm independence from the sponsors; the content of the article has not been influenced by the sponsors.

## AUTHOR CONTRIBUTIONS

Magdalena Cerón‐Rodríguez contributed in conception, design, analysis of data, interpretation of data and review. Edgar Barajas‐Colón contributed in conception, analysis of data, interpretation of data and review. Guillermo Ramón‐García contributed in analysis of data, interpretation of data and review. Isidro Franco‐Álvarez contributed in analysis of data, interpretation of data and review. Juan L Salgado‐Loza contributed in conception, design, analysis of data, interpretation of data and drafting.

## ETHICAL APPROVAL

It was not required. A patient consent statement for all articles or other material that contain personal information about a patient was obtained. No animals were used in this article.
